# Whole genome sequencing and characteristics of extended-spectrum beta-lactamase producing *Escherichia coli* isolated from poultry farms in Banaskantha, India

**DOI:** 10.3389/fmicb.2022.996214

**Published:** 2022-10-14

**Authors:** Mitul A. Patel, Aparna Pandey, A. C. Patel, S. S. Patel, H. C. Chauhan, M. D. Shrimali, Pankaj A. Patel, S. K. Mohapatra, B. S. Chandel

**Affiliations:** ^1^Department of Biotechnology, Sankalchand Patel University, Visnagar, India; ^2^Department of Biochemistry, Dental College, Sankalchand Patel University, Visnagar, India; ^3^Department of Veterinary Microbiology, Veterinary College, Kamdhenu University, Sardarkushinagar, India; ^4^Department of Animal Biotechnology, Veterinary College, Kamdhenu University, Sardarkushinagar, India; ^5^Department of Physiology, Veterinary College, Kamdhenu University, Sardarkushinagar, India

**Keywords:** whole genome sequencing, ESBL, *Escherichia coli*, antimicrobial resistance, plasmids, polymerase chain reaction, SNPs

## Abstract

Worldwide dissemination of extended-spectrum -lactamase (ESBL)-producing *Escherichia coli* constitutes an emerging global health issue, with animal food products contributing as potential reservoirs. ESBL *E. coli* infection is associated with the high mortality and mobility rate in developing countries due to less susceptibility to antibiotics. The present study aimed to elucidate the molecular characteristics and sequence-based analysis of ESBL *E. coli* in the Gujarat state of India. This study included 108 *E. coli* strains were isolated from different poultry farms (broiler and layer) in the Banaskantha District. PCR was employed to identify genotypic ESBL-producing antimicrobial resistance genes. Overall, a high occurrence of ESBL genes was found in poultry farms due to the high usage of antimicrobials. The PCR analysis revealed that 79.62% of isolates were detected positive with one or more ESBL genes. Among them, *bla*_TEM_ (63.88%) was found to be the predominant genotype, followed by *bla*_SHV_ (30.55%) and *bla*_OXA_ (28.70%). In the *bla*_CTX-M_ group, a higher occurrence was observed in *bla*_CTX-M-9_ (23.14%), followed by *bla*_CTX-M-2_ (24.07%) and *bla*_CTX-M-1_ (22.22%). We used the whole-genome sequencing (WGS) method to evaluate the antimicrobial resistance genes, virulence factors, single nucleotide polymorphisms (SNPs), plasmid replicons, and plasmid-mediated AMR genes of one ESBL *E. coli* isolated. We examined the genetic relatedness of a human pathogenic *E. coli* strain by comparing its sequence with the broad geographical reference *E. coli* sequences. *Escherichia coli* ST 681 was determined using multi-locus sequence typing. We compared our findings to the reference sequence of *Escherichia coli* str. K- 12 substr. MG1655. We found 24,937 SNPs with 21,792 in the genic region, 3,145 in the intergenic region, and six InDels across the genome. The WGS analysis revealed 46 antimicrobial resistance genes and seven plasmid-mediated AMR genes *viz.*, *tetA*, *qnrS1, dfrA14*, *sul2*, *aph(3”)-lb*, *aph(6)-ld*, and *Aph(3’)-la*. The ST 681 was found to have *Cib*, *traT*, and *terC* virulence factors and two plasmid replicons, IncFII(pHN7A8) and IncI1-I(Alpha). This study revealed a higher occurrence of ESBL *E. coli* detected in poultry.

## Introduction

In recent years, bacterial resistance has been a global health concern. By 2050, the annual mortality toll from bacterial resistance may reach 10 million (O’Neill, 2014), with approximately 90% of predicted deaths happening in Asia and Africa (Islam et al., 2019). The development of bacterial resistance in humans and animals is significantly influenced by excessive use, inappropriate dosages, and inadequate treatments (Rahman et al., 2020). Resistance is rapidly increasing by the spread of resistance genes using horizontal gene transfer or mobile genetic element mechanisms (Tzouvelekis et al., 2012; Partridge et al., 2018). Various aspects, such as insertion sequence (IS) elements, bacteriophages, transposons, and plasmids, play a crucial role in the dissemination of antimicrobial resistance (Carattoli, 2013; Zeng et al., 2019). On the other hand, the bacterial genome continuously undergoes multiple mutations in a short time and harbors new antibiotic resistance genes, leading to the formation of a new pathogen (Herring et al., 2006). To address this context, advanced molecular technology microbial whole genome sequencing (WGS) has emerged as a tool that can be used to study the mutation rate, the discovery of new resistance genes, and the evolution of antimicrobial resistance using historical isolates (Koser et al., 2014). WGS is fast, incredibly accurate, and has become the gold standard technique for determining relatedness between strains and surveillance of food-borne pathogens (Rokney et al., 2020; Moghnia and Sweih, 2022).

*Escherichia coli* (*E. coli*) is not only an intestinal and extraintestinal disease-causing pathogen but also a significant contributor to antimicrobial resistance (AMR). According to AMR surveillance systems, *E. coli* bacteria are often carriers of extended-spectrum beta-lactamase genes (ESBL) genes, making them resistant to major antibiotic classes (Pitout, 2012). In recent years, significant increases of beta-lactamase in gram-negative bacteria and a higher occurrence of *bla*_TEM_, *bla*_SHV_, *bla*_OXA_, and *bla*_CTX-M_ group 1, 2, and 9 have made the situation worrying. The occurrence of infections due to extended-spectrum beta-lactamase-producing (ESBL) bacteria has increased globally in both community and healthcare-associated contexts (Manges et al., 2019). Worldwide, poultry is the potential reservoir for spreading ESBL-producing *E. coli* to humans by direct contact or consuming contaminated meat products (Aworh et al., 2020). Studying the genetic components of antimicrobial resistance genes is essential for controlling and interpreting the patterns of changing resistance among bacteria (Deter et al., 2021).

In India, the poultry industry is one of the fastest-growing segments in the agricultural sector today. India is the third-largest producer of eggs in the world, behind China and the United States, and the seventh-largest chicken meat producer (FAO, 2014). Like many other developing countries, maintaining hygienic conditions in poultry farms and raw food processing plants is still underdeveloped. There are limited antimicrobial resistance surveillance studies of farm animals in India. The repeated use of low doses of antibiotics as growth promoters and prophylactic chemotherapy in chickens creates the perfect environment for developing and transmitting AMR in animal and human populations (You and Silbergeld, 2014).

The feces were the source of the dissemination of multidrug-resistant *E. coli* from poultry to the human population (Sebastian et al., 2021; Day et al., 2019). Therefore, the present study aims to identify the genotypic resistance pattern of ESBL *E. coli* from poultry fecal samples by screening ESBL genes (*bla*_TEM_, *bla*_SHV_, *bla*_OXA_, and *bla*_CTX-M_ group 1, 2, and 9). We used whole genome sequencing (WGS) to understand the genetic variations among the multidrug resistance (MDR) *E. coli* isolated from poultry fecal samples and compared them to other reported genomes retrieved from the NCBI database to determine their relatedness. Additionally, it sought to identify the molecular characteristics of the AMR genes, virulence factors, SNPs, and plasmid replicon of ESBL-producing *E. coli* isolated from poultry fecal samples.

## Materials and methods

### Sample collection

One hundred twenty cloacal swabs were randomly collected from 30 different poultry farms (four from each farm) during year of 2021 in Banaskantha District. Followed aseptic precautions by gently inserting a sterile swab into the chicken’s cloaca and carefully swabbing it on the mucosal wall several times. Samples were packed carefully and transported immediately to a laboratory at 4°C. Samples were collected from those birds which were going to enter the food chain and were considered to be sold for human consumption. Dead, sick, and treated with antibiotics in the previous week were excluded from the study.

### Bacteria isolation

Primary isolation was carried out by inoculating the cloacal swab into MacConkey broth for pre-enrichment and incubated at 37°C for 24 h. Then the incubated culture was streaked on MacConkey agar and incubated at 37°C for 24 h. followed by Eosin Methylene Blue (EMB) agar for selective isolation; the plate was incubated at 37°C for 24 h. The microscopic morphology of the isolates was studied by Gram’s staining method, followed by biochemical tests like Oxidase, catalase, Indole, Methyl red, Voges-Proskauer, and citrate were employed to confirm *E. coli* as per the methods described by Ewing (1972).

### DNA extraction, identification, and phenotypic analysis

The genomic DNA of the bacterial culture was extracted by the QIAamp DNA mini kit (QIAGEN). Bacterial DNA extraction was carried out according to the instruction booklet provided with the kit. The quality and quantity of extracted genomic DNA were determined using a picodrop spectrophotometer (Picodrop Ltd., Cambridge, United Kingdom). The purity of the DNA sample was further verified by running the DNA sample in 0.8 percent of the agarose gel in electrophoresis.

*Escherichia coli* isolates were identified through the amplification of the species-specific 16S rRNA gene (ECO-1) using primers listed in Table 1. The PCR reaction was conducted with 12.5 μl master mix (Qiagen), 8.5 μl nuclease-free water, 1 μl of each primer, and 2 μl of DNA template. All the PCR reactions were performed for 30 cycles in a 25 μl final reaction mixture. The amplification conditions were as follows: initial denaturation at 94°C for 5 min, followed by 30 cycles of denature at 94°C for 30 s, annealing at 53°C for 1 min, an extension at 72°C for 1 min, and a final extension at 72°C for 8 min (Fratamico et al., 2000).

**Table 1:** Details of primers for amplification of ESBL *bla*_TEM_, *bla*_SHV_, *bla*_OXA_, *bla*_CTX-M-1_, *bla*_CTX-M-2_, *bla*_CTX-M-9_, and ECO-I genes.

Gene designated	Primer sequence (5′- 3′)		Size of amplified products (bp)	References
*bla*_CTX-M-1_	F	TTAGGAARTGTGCCGCTGYA	688	Dallenne et al. (2010)
	R	CGATATCGTTGGTGGTRCCAT		
*bla*_CTX-M-2_	F	CGTTAACGGCACGATGAC	404	
	R	CGATATCGTTGGTGGTRCCAT		
*bla*_CTX-M-9_	F	TCAAGCCTGCCGATCTGGT	561	
	R	TGATTCTCGCCGCTGAAG		
*bla*_TEM_	F	CATTTCCGTGTCGCCCTTATTC	800	
	R	CGTTCATCCATAGTTGCCTGAC		
*bla*_SHV_	F	AGCCGCTTGAGCAAATTAAAC	713	
	R	ATCCCGCAGATAAATCACCAC		
*bla*_OXA_	F	GGCACCAGATTCAACTTTCAAG	654	
	R	GACCCCAAGTTTCCTGTAAGTG		
*bla*_ECO-1_	F	GACCTCGGTTTAGTTCACAGA	585	Fratamico et al. (2000)
	R	CACACGCTGACGCTGACCA		

For phenotypic confirmation of ESBL production, all the *E. coli* isolates detected by PCR were forwarded for phenotypic confirmation of ESBL-producing *E. coli* using a combined disc method phenotypic confirmation test kit (HiMedia, India). The test used *K. pneumoniae* (ATCC-700603) as a positive control and *E. coli* (ATCC- 25922) as a negative control. The results were interpreted as per the CLSI guidelines (2018).

### Identification of β-lactamase producing genes

All the *E. coli* isolates were screened for β-Lactamase producing genes (*bla*_TEM_, *bla*_SHV_, *bla*_OXA_, and *bla*_CTX-M_ group 1, 2, and 9) by PCR using various primers listed in Table 1. The amplification of DNA was performed within the Applied Biosystem thermocycler. A final reaction mixture was set at 25 μl. The amplification condition was as follows: initial denaturation at 94°C for 10 min, followed by 30 cycles of denature at 94°C for 40 s, annealing at 60°C for 40 s, an extension at 72°C for 1 min, and a final extension at 72°C for 7 min (Dallenne et al., 2010). Amplified products were analyzed on 2% agarose gel at 80 V/cm for 60 min.

### Whole genome sequencing

Genomic DNA was extracted from one multidrug-resistant isolate, ESBL *E. coli*, using the Quick-DNA Miniprep Plus Kit (Zymo Research) according to the manufacturer’s protocol. The concentration of DNA was determined by a picodrop spectrophotometer. Isolate sequencing libraries were prepared by the Illumina TruSeq Nano DNA Library Prep Kit (Illumina) according to the manufacturer’s protocol. The quality and quantity of the library was checked using the Agilent Technologies 4200 Tape Station system. Following that, Illumina sequencing was performed using the NextSeq500 platform to generate 2 × 150 bp paired-end reads. The sequence of the isolate has been submitted to the NCBI database under BioProject PRJNA846119, and the assigned GenBank accession number is NZ_CP098739.1.

### Genome assembly

To filter raw reads, paired-end data were collected in fastq file format. FastQC v0.11.7 was used to verify the quality of sequence reads before and after quality filtering. Trimmomatic v0.38 was used to trimming adapter sequences. Ambiguous reads (reads with unknown nucleotides “N” larger than 5%), and low-quality sequences [reads with more than 10% quality threshold (QV) < 20 Phred score; Bolger et al., 2014]. Reads were mapped using BWA MEM (version 0.7.17; Burrow- Wheeler Aligner; Li and Durbin, 2009).

### Single nucleotide polymorphisms (SNPs) identification and genome characterization

The mpileup utility of Samtools (v 0.1.18; Li and Durbin, 2009) was used to identify SNPs and InDels from the sorted BAM file of each mapping. The variants were filtered based on a minimum read depth of 15 and a quality threshold of 25. The identified variants were annotated using the bedtools intersect tool. The complete genome of *E. coli* K12-MG1655 was used as a reference genome.

For the identification of acquired antimicrobial resistance genes, the Comprehensive Antimicrobial Resistance Database (CARD; Liu and Pop, 2009) was used with the following settings: a selected ID threshold of 95% and a selected minimum length of 60%. The genome sequence of the isolate was aligned against VirulenceFinder 2.0 to predict virulence genes (Joensen et al., 2014) and against ResFinder 4.1 to detect plasmid-mediated ARGs with a nucleotide identity threshold of 60% and a minimum length. PlasmidFinder 2.1 was used with default settings to determine plasmid types in an isolated sequence.

### Detection of serotyping and phylogenetic analysis

The KU_Poultry_13’s serotype and sequence type (ST) were determined using MLST 2.0 (Larsen et al., 2012) and a SerotypeFInder 2.0 (Joensen et al., 2015) with settings of min. 5X Depth for an allele and selected ID threshold of 90% and a selected minimum length of 60%, respectively.

For phylogenetic analysis, the reference sequences of WGS were sourced from PATRIC 3.6.12 and the NCBI database. A whole genome-based phylogenetic analysis was performed using the Type Stain Genome Server (TYGS) as described previously (Lagesen and Hallin, 2007; Meier-Kolthoff and Goker, 2019). TYGS offers a pairwise similarity calculation and a typical phylogenetic technique that includes multiple sequence alignment and analysis using maximum-likelihood and maximum-parsimony criteria. The Genome BLAST Distance Phylogeny (GBDP) technique may also use TYGS to quickly infer trees with branch support values, allowing for determining dDDH values. A reference sequence was included based on year, country, and host metadata to reveal a close relatedness of the sample sequence with other reference sequences and support the placement of Indian sequences as an international reference. The phylogeny was visualized and annotated on the web-based iTOL platform (Letunic and Bork, 2019).

## Results

### Detection of *Escherichia coli* and ESBL-producer

**Figure 1:**
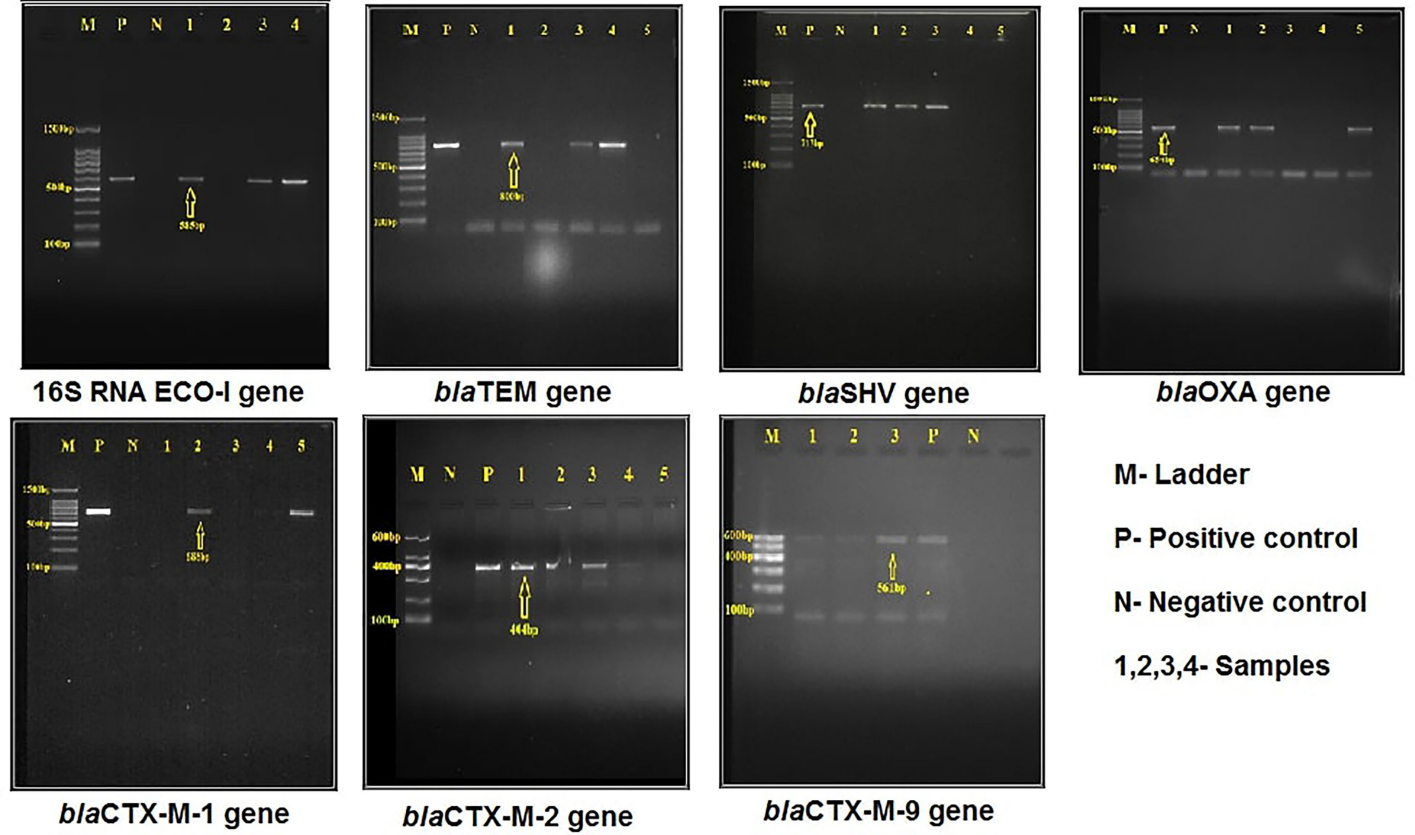
Detection of species-specific and major extended-spectrum –lactamase (ESBL)-producing genes in *Escherichia coli*.

The samples giving positive results for the 16S rRNA gene with an amplicon size of 585 bp were confirmed as *E. coli* (Figure 1). 108 samples out of 120 were confirmed positive for *E. coli*; among them, half of the samples were confirmed as phenotypic ESBL producers of *E. coli*.

### PCR based detection of major β-lactamase genes of *Escherichia coli*

PCR analysis revealed that 86 (79.62%) isolates were positive for one or more ESBL-producing genes (Figure 1). Among them, 69 (63.88%) isolates tested positive for the *bla*_TEM_ gene, with broiler 40 (37.03%) and layer 29 (26.85%) showing the highest occurrence and correlated with the most phenotypic resistance gene. Thirty-three (30.55%) isolates were discovered positive for the *bla*_SHV_ gene with broiler (17.59%) and layer (12.96%) of the total. The *bla*_OXA_ gene was confirmed to be positive in 31(28.70%) samples, with broiler 19 (17.59%), and layer 12 (11.11%) of the total.

**Figure 2:**
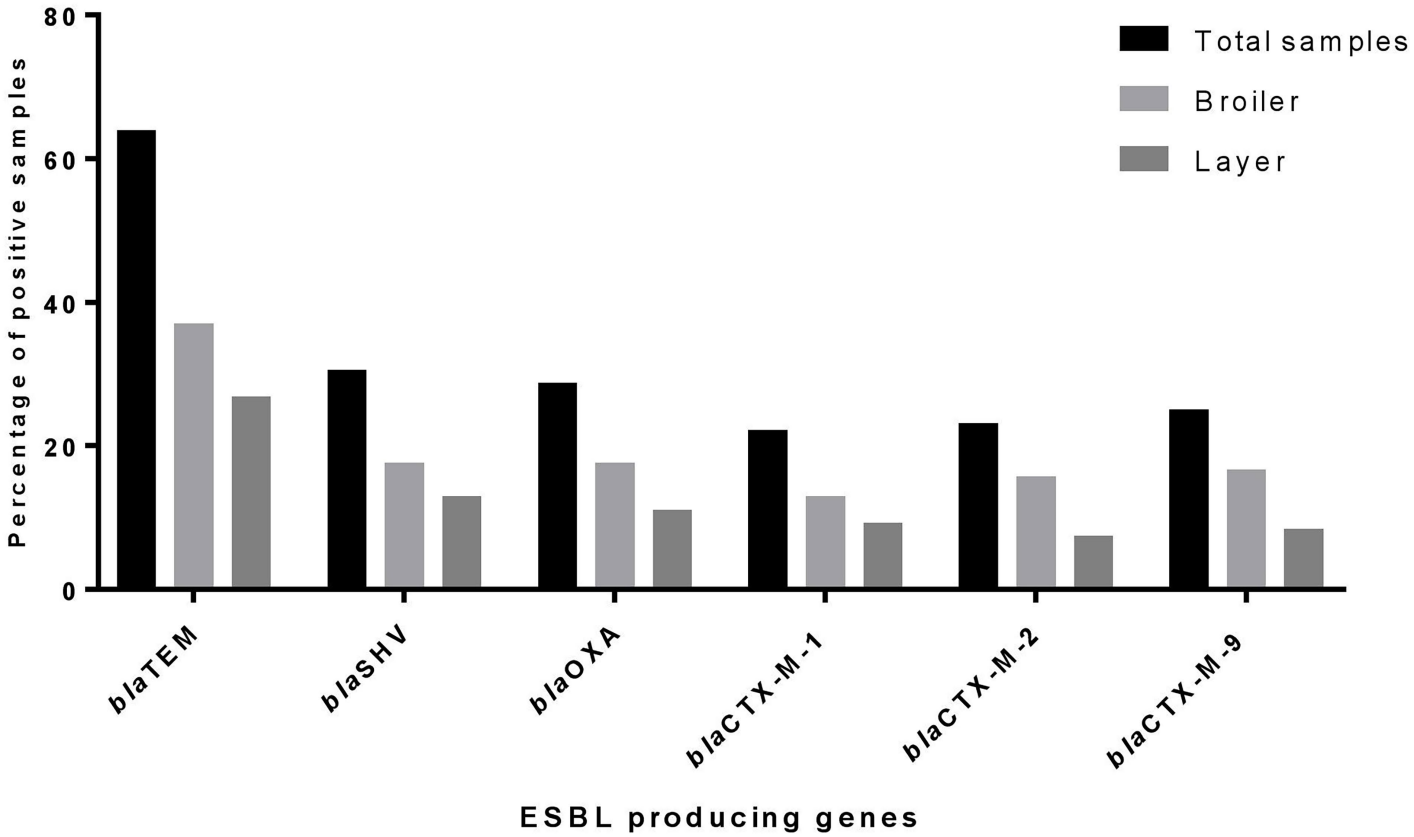
Distribution pattern of major ESBL-producing genotype.

In the blaCTX-M group, 24 (22.22%) samples were identified to be positive for the blaCTX-M-1 gene, with broiler 14 (12.96%) and layer 10 (9.26%) of the total. The blaCTX-M-2 gene was positive in 26 (24.07%) isolates, with broiler 17 (15.74%) and layer 9 (8.33%) of the total. The presence of the blaCTX-M-9 gene was detected in 25 (23.14%) isolates, with broiler 17 (15.74%) and layer 8 (7.40%) of the total. The details of sample-wise detection of ESBL genes are mentioned in Supplementary Table S1 (Figure 2), and the amplicon size of each gene was described in Table 1.

The occurrence of primary ESBL-producing genes among isolates revealed that 42 isolates contain two or more ESBL genes. The co-existence of blaTEM, blaSHV, and blaOXA was observed in 12 (28.57%) isolates, while blaTEM and blaOXA were detected in 15 (35.71%) isolates. The 12 (28.57%) and 3 (7.14%) isolates were identified in combination with blaTEM with blaSHV and blaOXA with blaSHV genes, respectively. Additionally, 16 isolates were detected with a combination of blaCTX-M group genes. The co-existence of blaCTX-M-1 and blaCTX-M-9 was identified in one (6.25%) of the isolates, while 3 (18.75%) of the isolates were detected with blaCTX-M-2 and blaCTX-M-9 combination. A higher occurrence was observed with the co-existence of blaCTX-M-1 and blaCTX-M-2 genes in 11 (68.75%) isolates.

### Single nucleotide polymorphisms and InDels identification of isolate

To identify the mutation rate and distinction of very closely related organisms as very limited allelic differences on similar genomic structures, such as different serotypes of *E. coli*, which may be causing the pathogenicity of organisms and their risk of developing diseases. The isolated sequence was annotated using the bedtools intersect tool. Mapping to the reference genome, *Escherichia coli* str. K-12 substr. MG1655 (NC_000913.3), a total of 24,152 SNPs were identified in the isolate sequence; among them, 21,792 SNPs were located in the genic region, and the total number of InDels identified was 6 (Table 2), of which 1 Indel (Table 3), was located in the genic region.

**Table 2:** Total number of InDels identified.

Chromosom e	Position	Reference Allele	Alternate Allele	Read Depth	Mapping Quality	GenoType	Format	Description
NC_000913.3	228,687	ATT	AT	147	29	Homozygous	GT:PL:GQ	1/1:210,255,0:99
NC_000913.3	357,674	TG	T	72	34	Heterozygous	GT:PL:GQ	0/1:63,0,167:66
NC_000913.3	781,853	C	CT	195	55	Homozygous	GT:PL:GQ	1/1:255,255,0:99
NC_000913.3	2,035,390	GCCCC	GCCCCCC,GC CCCCCC	211	60	Heterozygous	GT:PL:GQ	1/1:95,29,17,73, 0,70:21
NC_000913.3	3,808,174	GAAAAAA	GAAAAA	151	60	Homozygous	GT:PL:GQ	1/1:81,39,0:73
NC_000913.3	4,549,760	A	AC	209	60	Homozygous	GT:PL:GQ	1/1:152,42,0:81

**Table 3:** Identified InDels located in genic region.

Chromosome	Position	Reference Allele	Alternate Allele	Gene Name	Gene Start	Gene End	Description
NC_000913.3	3,808,174	GAAAAAA	GAAAAA	b4760	3,808,166	3,808,238	Small regulatory RNA RirA

### Detection of antimicrobial resistance genes

Using the CARD database, we identified 46 antimicrobial resistance genes to various classes of antibiotics. The details of each antibiotic resistance gene and their associated AMR gene family, antimicrobial class, and resistance mechanism are provided in Supplementary Table S2. Seven plasmid-mediated ARG genes were detected in the isolate sequence using ResFinder 4.1. Isolate stain was resistance to tetracycline, quinolone (*qnrS1*), trimethoprim (*dfrA14*), sulfamethoxazole (*sul2*), streptomycin *[aph(3″)-lb][aph(6)-ld]*, aminoglycoside *[Aph(3′)-la]* and their identity, position in contigs, and phenotype are described in Table 4.

**Table 4:** Identification of Plasmid mediated AMR genes in ESBL *E. coli* sequence.

Sr. No.	Category	AMR genes	Identity %	Alignment length	Position in contigs	Phenotype	Accession Number
1	Quinolone	qnrS1	100	657/657	4197038.4197694	Ciprofloxacin	AB187515
2	Tetracycline	Tet(A)	99.91	1200/1200	4191184.4192383	Doxycycline/ tetracycline	AJ517790
3	Folate pathway antagonist	Sul2	100	816/816	3478115.3478930	Sulfamethoxazole	AY034138
4		dfA14	100	474/474	3479539.3480012	Trimethoprim	AF393510
5	Aminoglycoside	aph(3″)-lb	100	529/804	3478991.3479519	Streptomycin	AF321551
6		aph(6)-ld	99.86	726/837	3478991.3479519	Streptomycin	AF024602
7		Aph(3′)-la	100	816/816	4742471.4743286	Neomycin,Kanamycin,lividomycin, paromomycin, ribostamycin	V00359

### Identification of acquired virulence genes, plasmid, and phylogenetic analysis

The sequence of the isolate harbors four virulence genes, *viz.*, *Cib*, *traT*, and two *terC*. The *cib*, colicon ib. gene was found in the sequence. A *traT* gene code for an outer membrane protein linked to serum resistance and ExPEC, and two *terC* genes code for tellurium ion resistance protein. The details of each virulence gene’s identity, template length, and contig position were mentioned in Table 5. The PlasmidFinder 2.1 was applied to determine plasmids in the whole genome sequence. Two plasmid sequences, IncFII (pHN7A8) and IncI1-I(Alpha), were identified from *E. coli* isolates. IncFll plasmid, which is a low copy number plasmid. The Incl-1 plasmid is commonly found in enteric bacteria in food and animal sources. The details of the plasmid with identity, template length, contigs, and position of contigs are provided in Table 6.

**Table 5:** Detection of virulence genes in ESBL *E. coli* sequence.

Sr. No.	Virulence genes	Identity	Template length	Contigs	Position in contigs	Protein function	Accession Number
1	*cib*	100	1,881/1,881	contig_101	7132.9012	Colicin ib	HQ114281
2	*terC*	100	714/714	contig_73	17935.18648	Tellurium ion resistance protein	CP007491
3	*terC*	99.9	959/966	contig_20	30583.31541	Tellurium ion resistance protein	MG591698
4	*traT*	100	777/777	contig_60	23483.24259	Outer membrane protein complement resistance	UCYO01000010

**Table 6:** Detection of Plasmids in ESBL *E. coli* sequence.

Sr. No.	Plasmid	Identity	Query/Template length	Contig	Position in contig	Accession number
1	IncFII(pHN7A8)	100	260/260	KU_Poultry_13_(paired)_contig_60	36824.37083	JN232517
2	IncI1-I(Alpha)	100	142/142	KU_Poultry_13_(paired)_contig_84	15896.16037	AP005147

**Figure 3:**
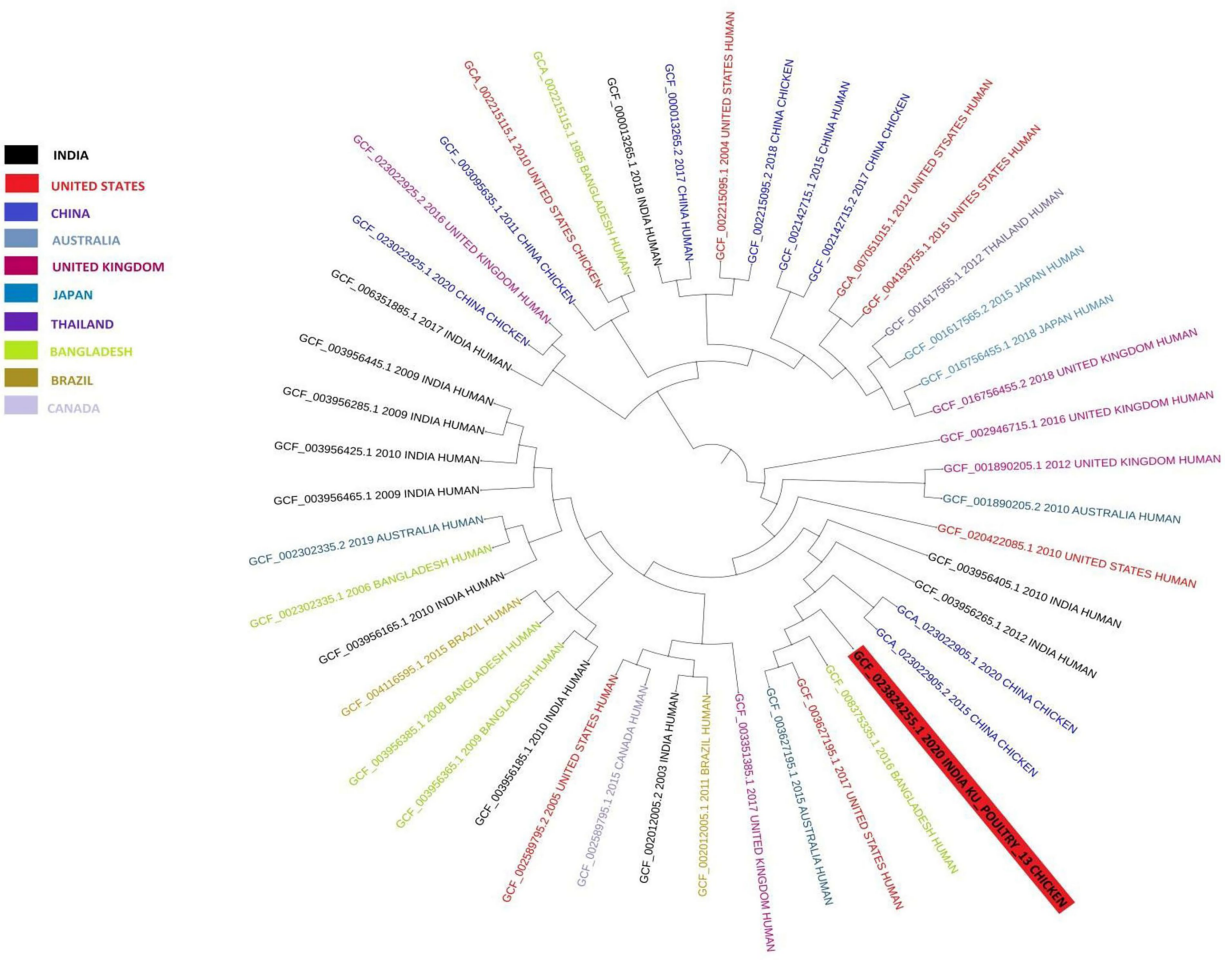
The maximum likelihood phylogenetic tree showing genetic relateness of *Escherichia coli* strain KU_POULTRY_13_ECOLI. A total of 46 *E. coli* strains were used from different geographic locations, including publicly available *E. coli* strains. Tree inferred with FastME 2.1.6.1 from Genome BLAST Distance Phylogeny (GBDP) distances calculated from genome sequences branch lengths are scaled in terms of GBDP distance formula d5. The numbers above branches are GBDP pseudo-bootstrap support values >60% from 100 replications, with an average branch support of 46.8%. The tree was rooted at the midpoint.

The KU_Poultry_13 *E. coli* was determined to be ST 681 and serotype O40:H4. A maximum likelihood phylogenetic tree (Figure 3) of the poultry fecal sample KU_Poultry_13 *E. coli* sequence was performed to compare the whole genome sequences of known Indian human and other countries’ human and chicken *E. coli* strains were analyzed in this study from broad geographical locations, including the United States, Japan, Canada, the United Kingdom, Bangladesh, Australia, China, Thailand, and Brazil. There are two well-supported broad clades on the phylogenetic tree, which are well-supported subclades. The KU_Poultry_13 *E. coli* sequence from poultry is located far from the root in a small, internationally diversified clade made up of strains from different periods. It was closely related to the human-sourced stains GCF_003956265.1 from India (2012), GCF 008375335.1 from Bangladesh (2016), and GCF 003627195.1 from the United States (2017). It was also closely related to the GCA_023022905.1 from China (2020) chicken sourced and the GCF_003956405.1 from India (2012) human-sourced. Another Indian human stain from 2017 and 2018 was placed in separate major clades and had no very close relatives.

## Discussion

Extended-pectrum beta-lactamase produces *E. coli* are increasingly important sources of -lactam antibiotic resistance and have significant repercussions for efficiently managing bacterial infections (Abike et al., 2018). Limited genomic studies have been done regarding the occurrence of ESBL-producing *E. coli* from poultry fecal material in India. The current study was conducted to determine the antimicrobial resistance genes’ status in ESBL-producing *E. coli* from poultry fecal samples. We employed the WGS method to identify virulence genes, SNPs, and antimicrobial resistance genes (including plasmid-mediated) and also carried out evolutionary studies.

The genotype of our isolates (ESBL and non-ESBL) revealed that a total of 86 (79.62%) isolates were positive for one or more ESBL-producing genes. We found a high occurrence of *bla*_TEM_ (63.88%), which is associated with a major phenotypic resistance-producing gene in *E. coli*. Our result showed a higher occurrence of *bla*_TEM_ compared to previous studies (Kahraman et al., 2016; Sharma et al., 2021) in ESBL-producing *E. coli* isolates derived from poultry fecal samples. The present study revealed the occurrence of *bla*_SHV_ and *bla*_CTX-M-1_ genes is 30.55 and 22.22%, respectively, among isolates, which is in line with previously published reports (Andy et al., 2019; Gundran et al., 2019).

Several reports on multi-drug resistance ESBL producing *E. coli* have been thoroughly documented in healthy broilers (Li et al., 2016), animal sources (He et al., 2013), human clinical samples (Hassan and Abdalhamid, 2014), and a urine sample (Jena et al., 2017). In the current study, a fecal sample was processed for bacterial isolation and identification. The most prevalent combination in this investigation was the blaCTX-M gene with the blaTEM gene, with or without blaSHV, which is inconsistent with a previous report discovering these three genotypes in poultry cloacal swab samples from France (Selma et al., 2018). The co-existence of blaTEM + blaSHV + blaCTX-M was found in 6 (5.55%) isolates, and a combination of blaTEM and blaSHV showed an occurrence of 11.11% in the current study, which is in line with the findings of Masoud et al. (2021) in Egypt. The present study observed a significant association between blaCTX-M, blaTEM, and blaSHV. These data corroborate the descriptions made by Mohamed et al. (2020), Zaki et al. (2019), and Maleki et al. (2018).

The primer set we used in the current investigation identifies ESBL and non- ESBL *E. coli*. Out of 54 phenotypically non-ESBL *E. coli*, 31 (28.70%) isolates were detected with ESBL-producing genes using the PCR technique. Similar to our findings, the study from Central India reported the detection of β-lactamase producing genes in non-ESBL in *E. coli* and *K. pneumonia* from humans (Bajpai et al., 2017) and shrimp aquaculture farm in Kerala (Sivaraman et al., 2021). They were designated as phenotypically non-ESBL because they did not follow the CLSI standards for ESBL confirmation. Thirty-one non-ESBL *E. coli* carried one or more ESBL genes in the present study, *viz.*, *bla*_TEM_, *bla*_SHV_, *bla*_OXA_, or *bla*_CTX-M_ group; we cannot completely rule out the possibility of some of the ESBL genes identified here being non-ESBL type, but this might be due to the production of chromosomal or plasmid-mediated AmpC beta-lactamases can hide the presence of ESBL genes. As a result, ESBL-producing *E. coli* that contain AmpC beta-lactamases can cause false-negative ESBLs. The detection of ESBL genes in 28.70% of non-ESBL producing *E. coli* showed higher sensitivity to the genotypic method than the phenotypic confirmation test.

We used the WGS method to characterize one multi-drug resistant ESBL-producing *E. coli* isolated from a poultry fecal sample. We used MLST 2.0 to determine the sequence type (ST) of the selected *E. coli* as KU_Poultry_13 *E. coli* strain. The strain ST681 is reminiscent of the pathogenic ExPEC strains and lineages that cause infections in humans and animals (Bonnedahl et al., 2009).

The CARD and ResFinder 4.1 databases were used to analyze antimicrobial resistance genes. On the other hand, CARD came out on top with the largest number of prediction genes (both mutation and acquired-based resistance genes). The CARD database identified 46 resistance genes, more than the ResFinder database in the present study. As Xavier et al. (2016) reported, CARD conducted superior analysis utilizing whole-genome sequencing data than other databases such as ResFinder and CBMAR and reported the highest number of correct predictions. All 30 antimicrobial resistance genes originating from *E. coli* (*acrD,acrE, acrF, gadX, emrX, CRP, AcrS H-NS, mdtA, mdtB, mdtC, mdtE, mdtF, baeR, baeS, cpxA, evgA, evgS, mdtM, mdtK, mdtH, mdtG, mdtO, mdtP, and lptD*) were involved in the antibiotic efflux pump were identified in present study. Similar investigation findings were reported by Cudkowicz and Schuldiner (2019) in Israel. These genes have been previously described in this species, and many of them as even exclusive to it, according to the CARD database (Toth et al., 2021;Jia et al., 2016; McArthur et al., 2013). Gram-negative bacteria typically display the porins in their outer membrane involved in outer membrane protein A (*ompA*) that are responsible for reducing the permeability to antibiotics (Smani et al., 2014). Similar to our findings, *OmpA* was initially identified in 1974 by Foulds and Chai (1978) as a heat-modifiable protein in *E. coli*. The antibiotics’ target alteration genes, such as *bacA, eptA, udg*, and *pmrF* genes, were detected in the current study.

Our study identified two plasmid replicons, IncFII(pHN7A8) and IncI1- I(Alpha), in the strain. The *bla*_CTX-M_ genes are mostly linked to IncFII or IncI1 plasmids (Carattoli, 2009; Cao et al., 2011). The Incl1 plasmid was a potential source of carrying the *bla*_CTX-M-1_ gene and has been identified all over Europe (Hall et al., 2014). Similarly, in our study, the presence of the Incl1 plasmid carrying of *bla*_CTX-M-1_ gene was confirmed by the PCR. Plasmid-mediated AMR genes were identified using the ResFinder 4.1 database, are *tet(A)*, *qnrS1*, *dfrA14*, *sul2*, *aph(3″)-lb*, *Aph(3′)-la*, and *aph(6)-ld*. These genes are regulated by three resistance mechanisms: antibiotic inactivation, efflux pump, and antibiotic target alternation. The inactivation of aminoglycoside antibiotic is conferred by *aph(3″)-Ib* and *aph(6)-Id*, which is responsible for regulating aminoglycoside phosphotransferase and plays a significant role in aminoglycoside resistance, is the most common resistance mechanism known (Hua et al., 2020). Acquired sulfonamide resistance was first identified in the 1960s, but *sul1* and *sul2* plasmid-mediated genes were subsequently identified in the 1980s. These *in silico* findings give insights into *E. coli*, harboring resistance genes raises concern about antimicrobial resistance in isolated *E. coli* from fecal samples. These pathogens then enter into surroundings causing the spread of AMR and posing a great risk to animal and human health.

Founou et al. (2022) reported virulence gene ranging from 1 to 18 whereas we have detected four virulence genes in the genome sequence of our study presented in Table 5. All virulence genes detected by *in silico* analysis are also detected by Founou et al. (2022). The outer membrane exclusion protein *traT* mostly occur in ESBL producing *E. coli* from healthy food animal sources across Europe (Ewers et al., 2021) and in MDR *E. coli* in the Democratic Republic of Congo (Irenge et al., 2019). The cognate immune protein for colicin Ib (*cib* gene) inserts into the inner bacterial membrane, inhibiting Cib-mediated pore formation and death, as described by Cascales et al. (2007). Stecher et al. (2012) reported the detection of the *cib* gene in Enterobacteriaceae. The current investigation revealed the *cib* and *traT* genes, which is consistent with the results of the studies above-described. The tellurium ion-containing protein *terC* gene was detected in ExPEC *E. coli* (Tetzschner et al., 2020), which was also detected in our study.

The SNP-based technique offers a cost-effective alternative method for various bacterial species to understand the relatedness among different strains, including *E. coli* (Dias et al., 2010; Dallman et al., 2021). The WGS approach enables finding SNPs in bacteria using different bioinformatics tools. As a result, multiple polymorphisms may be utilized to see the variability between strains that are quite similar from different origins (Camprubi et al., 2018; Tzouvelekis et al., 2020). The current analysis revealed a total of 24,937 SNPs, with 21,792 in the genic region and 3,145 in the intergenic region, by comparing the reference whole genome sequence of *Escherichia* coli str. K-12 substr. MG1655 (NC_000913.3). In the 70 *E. coli* O157:H7 genomes, 3,313 SNPs were found, with 2,797 intragenic and 516 intergenic SNPs (Rusconi et al., 2016). In Canada, Parsons et al. (2016) observed that SNPs could be exploited to identify food-borne pathogens.

## Conclusion

*Escherichia coli* is a bacterium of normal gut flora of animals and humans, but it acquired antimicrobial resistance genes through horizontal gene transfer mechanisms. Poultry is the potential reservoir for spreading ESBL-producing *E. coli* to humans and animals by consuming chicken products in their food chain. It must be of appropriate concern for human as well as animal health. In the present study, we employed PCR and WGS-based technology to uncover the antimicrobial resistance, SNPs, and virulence genes profile of ESBL-producing *E. coli* strains in poultry. Our results indicate that ESBL-producing *E. coli* isolates from poultry fecal material contain several AMR genes, which are a potential source for dissemination to the environment, animals, and humans. Surveillance studies are necessary to monitor the rapid evolution of AMR genes from animals to humans. In the future, there is a need for a proper strategy for the containment of AMR and associated risk for human transmission.

## Data availability statement

The datasets presented in this study can be found in online repositories. The names of the repository/repositories and accession number(s) can be found in the article/Supplementary material.

## Author contributions

MP, HC, SP, and APan contributed to the hypothesis and design the experiment. MP and MS performed the sample collection. MP performed all the experiments and wrote the manuscript. MP, APat, SP, and SM analyzed the genotypic data. MP, APat, and APan analyzed the whole genome sequencing data, and PP created the figures and language correction. BC, APat, APan, and HC reviewed the manuscripts. All authors contributed to the article and approved the submitted version.

## Conflict of interest

The authors declare that the research was conducted in the absence of any commercial or financial relationships that could be construed as a potential conflict of interest.

## Publisher’s note

All claims expressed in this article are solely those of the authors and do not necessarily represent those of their affiliated organizations, or those of the publisher, the editors and the reviewers. Any product that may be evaluated in this article, or claim that may be made by its manufacturer, is not guaranteed or endorsed by the publisher.

## Footnotes

1. http://www.genomicepidemiology.org/services/
2. https://tygs.dsmz.de/
3. https://usegalaxy.org/
4. https://www.patricbrc.org/
