# Supplementary material for: Whole genome sequencing and characteristics of extended-spectrum beta-lactamase producing Escherichia coli isolated from poultry farms in Banaskantha, India
Source: Front Microbiol. 2022 Oct 14;13:996214. doi: 10.3389/fmicb.2022.996214 (PMC9614321; doi:10.3389/fmicb.2022.996214)
Supplement: Supplementary file 1 [file Table_1.DOCX]

**Table S 1 PCR based detection of ESBL producing genes of isolated *E. coli***

| **Sr. No** | **Sample**  **ID** | **TEM** | **SHV** | **OXA** | **CTX-M-1** | **CTX-M-2** | **CTX-M-9** |
| --- | --- | --- | --- | --- | --- | --- | --- |
| 1 | 1 | - | - | - | +VE | +VE |  |
| 2 | 2 | - | +VE | +VE | - |  |  |
| 3 | 4 | +VE | - | - | +VE | +VE | +VE |
| 4 | 6 | +VE | - | - | - | +VE | - |
| 5 | 7 | +VE | - | - | - | - | - |
| 6 | 8 | +VE | +VE | - | - | - | - |
| 7 | 9 | +VE | - | - |  | - | +VE |
| 8 | 11 | +VE | - | +VE | +VE | - | - |
| 9 | 12 | +VE | - | +VE | +VE | - | +VE |
| 10 | 13 | +VE | +VE | +VE | +VE | +VE | - |
| 11 | 14 | +VE | - | +VE | - | - | - |
| 12 | 15 | +VE | +VE | +VE | - | - | - |
| 13 | 16 | +VE | - | - | - | - | - |
| 14 | 17 | - | - | - | - | - | +VE |
| 15 | 18 | - | - | - | - | - | +VE |
| 16 | 19 | +VE | - | - |  | +VE | +VE |
| 17 | 21 | +VE | - | - | - | - | +VE |
| 18 | 22 | - | - | - | - | - | +VE |
| 19 | 24 | - | +VE | - | - | - | - |
| 20 | 25 | +VE | +VE | - | - | - | - |
| 21 | 26 | +VE | +VE | - | - | - | - |
| 22 | 27 | +VE | +VE | - | - | +VE | +VE |
| 23 | 30 | +VE | - | +VE | - | - | +VE |
| 24 | 31 | +VE | - | - | - | - | +VE |
| 25 | 32 | +VE | +VE | +VE | - | - | - |
| 26 | 33 | +VE | - | +VE | +VE | - | - |
| 27 | 34 | +VE | - | +VE | +VE | +VE | - |
| 28 | 35 | +VE | - | - | - | - | - |
| 29 | 36 | - | +VE | - | - | +VE | - |
| 30 | 37 | - | - | - | - | - | +VE |
| 31 | 38 | +VE | +VE | +VE | +VE | - | - |
| 32 | 39 | - | - | - | +VE | +VE | - |
| 33 | 40 | - | +VE | +VE | - | +VE | - |
| 34 | 42 | +VE | - | - | +VE | - | - |
| 35 | 45 | +VE | - | - | - | - | +VE |
| 36 | 46 | +VE | - | - | - | - | - |
| 37 | 47 | +VE | +VE | - | - | - | - |
| 38 | 48 | +VE | - | - | - | - | +VE |
| 39 | 50 | +VE | - | - | +VE | +VE | - |
| 40 | 51 | +VE | - | +VE | +VE | +VE | - |
| 41 | 52 | +VE | - | - | - | - | +VE |
| 42 | 55 | - | +VE | - | - | - | +VE |
| 43 | 56 | +VE | +VE | - | - | - | - |
| 44 | 57 | +VE | +VE | - | - | - | - |
| 45 | 58 | +VE | +VE | - | - | - | +VE |
| 46 | 62 | +VE | - | +VE | - | +VE | - |
| 47 | 63 | +VE | - | - | - | - | +VE |
| 48 | 64 | - | +VE | - | - | - | - |
| 49 | 67 | +VE | +VE | +VE | +VE | - | - |
| 50 | 68 | - | - | - | +VE | - | - |
| 51 | 69 | - | +VE | +VE | - | - | - |
| 52 | 71 | +VE | - | - | - | +VE | - |
| 53 | 72 | +VE | +VE | - | - | - | - |
| 54 | 73 | +VE | - | - | - | - | - |
| 55 | 74 | +VE | - | - | +VE | +VE | - |
| 56 | 75 | - | +VE | - | - | - | - |
| 57 | 77 | +VE | +VE | +VE | +VE | - | - |
| 58 | 78 | +VE | - | +VE | +VE | - | - |
| 59 | 80 | +VE | - | - | - | - | - |
| 60 | 81 | +VE | - | - | - | - | - |
| 61 | 82 | +VE | +VE | +VE | - | +VE | - |
| 62 | 84 | +VE | - | +VE | +VE | - | - |
| 63 | 86 | +VE | - | +VE | +VE | +VE |  |
| 64 | 89 | +VE | - | - | - | - | +VE |
| 65 | 92 | +VE | - | - | - | - | - |
| 66 | 94 | - | +VE | - | - | - | +VE |
| 67 | 96 | +VE | +VE | - | - | +VE | - |
| 68 | 97 | +VE | +VE | - | - | +VE | +VE |
| 69 | 98 | +VE | +VE | - | - | +VE | - |
| 70 | 102 | +VE | - | +VE | - | +VE | - |
| 71 | 103 | +VE | - | - | - | - | +VE |
| 72 | 104 | +VE | +VE | +VE | - | - | - |
| 73 | 105 | +VE | - | +VE | +VE | - | - |
| 74 | 106 | +VE | - | - | - | - | - |
| 75 | 108 | +VE | - | - | - | - | - |
| 76 | 110 | +VE | - | - | +VE | - | +VE |
| 77 | 111 | - | - | - | - | +VE | - |
| 78 | 112 | +VE | - | - | - | - | - |
| 79 | 113 | +VE | - | +VE | - | - | +VE |
| 80 | 114 | +VE | +VE | +VE | - | +VE | - |
| 81 | 115 | +VE | - | - | - | - | +VE |
| 82 | 116 | +VE | +VE | +VE | +VE | +VE | - |
| 83 | 117 | +VE | - | +VE | - | - | - |
| 84 | 118 | +VE | +VE | +VE | - | - | - |
| 85 | 119 | +VE | - | +VE | +VE | +VE | - |
| 86 | 120 | +VE | +VE | +VE | +VE | +VE | - |
